# Impact of outdoor residual spraying on the biting rate of malaria vectors: A pilot study in four villages in Kayin state, Myanmar

**DOI:** 10.1371/journal.pone.0240598

**Published:** 2020-10-29

**Authors:** Victor Chaumeau, Ladda Kajeechiwa, Thithiworada Kulabkeeree, Ramesh Kumar Vishwakarma, Praphan Wasisakun, Saw Nay Hsel, Kyi Oo, Tee Dah, Sunisa Sawasdichai, Muesuwa Trakoolchengkaew, Monthicha Phanaphadungtham, Aritsara Inta, Yanada Akararungrot, Naw Yu Lee, Prasan Kankew, Jacher Wiladphaingern, Mavuto Mukaka, Gilles Delmas, François Nosten

**Affiliations:** 1 Shoklo Malaria Research Unit, Mahidol-Oxford Tropical Medicine Research Unit, Faculty of Tropical Medicine, Mahidol University, Mae Sot, Thailand; 2 Nuffield Department of Medicine, Centre for Tropical Medicine and Global Health, University of Oxford, Oxford, United Kingdom; 3 Mahidol-Oxford Tropical Medicine Research Unit, Faculty of Tropical Medicine, Mahidol University, Bangkok, Thailand; Johns Hopkins University, UNITED STATES

## Abstract

Outdoor and early mosquito biters challenge the efficacy of bed-nets and indoor residual spraying on the Thailand-Myanmar border. Outdoor residual spraying is proposed for the control of exophilic mosquito species. The objective of this study was to assess the impact of outdoor residual spraying on the biting rate of malaria vectors in Kayin state, Myanmar. Outdoor residual spraying using lambda-cyhalothrin was carried out in two villages in December 2016 (beginning of the dry season) and two villages were used as a control. Malaria mosquitoes were captured at baseline and monthly for four months after the intervention using human-landing catch and cow-baited trap collection methods. The impact of outdoor residual spraying on human-biting rate was estimated with propensity score adjusted generalized linear mixed-effect regressions. At baseline, mean indoor and outdoor human-biting rate estimates ranged between 2.12 and 29.16 bites /person /night, and between 0.20 and 1.72 bites /person /night in the intervention and control villages respectively. Using model output, we estimated that human-biting rate was reduced by 91% (95%CI = 88–96, P <0.0001) immediately after outdoor residual spraying. Human-biting rate remained low in all sprayed villages for 3 months after the intervention. Malaria vector populations rose at month 4 in the intervention villages but not in the controls. This coincided with the expected end of insecticide mist residual effects, thereby suggesting that residual effects are important determinants of intervention outcome. We conclude that outdoor residual spraying with a capsule suspension of lambda-cyhalothrin rapidly reduced the biting rate malaria vectors in this area where pyrethroid resistance has been documented.

## Introduction

The Thailand-Myanmar border is an area where malaria transmission is low, seasonal and unstable [1]. *Plasmodium falciparum* was eliminated from most endemic villages with widespread deployment of community-wide access to early diagnostic and treatment with artemisinin-based combination therapies, and mass-drug administration campaigns in places where submicroscopic malaria prevalence was high [2]. Although the endemicity of vivax malaria has also declined in recent years [3], it is more difficult to tackle than falciparum malaria because of some features in the biology of *P*. *vivax* [4–6].

In this area, the primary mosquito vectors are *Anopheles minimus* (*s*.*s*.) (Minimus Complex, Funestus Group), *An*. *maculatus* (*s*.*s*.), *An*. *sawadwongporni* (Maculatus Group), *An*. *dirus* (*s*.*s*.) and *An*. *baimaii* (Dirus Complex, Leucosphyrus Group). *Anopheles pseudowillmori* (Maculatus Group), *An*. *aconitus* (*s*.*s*.) (Aconitus Subgroup, Funestus Group) and some members in the Annularis and Barbirostris Groups are secondary vectors [7–9]. Biting rate can be very high, thereby playing a disproportionate role in driving transmission intensity in this setting where *Plasmodium*-infection rates in mosquito populations are low [7, 10]. Bed-nets and indoor residual spraying fail to prevent most of malaria infections [11–13] because of the ecology and biology of relevant *Anopheles* species, including exophily and exophagy, zoophagy and opportunistic blood type selection, and activity peaks at dusk and dawn [7, 14, 15]. Larval source management is difficult to implement because of the diverse and fragmented nature of larval habitats [16], and because incredibly high densities of vector larvae can be found over large areas covered with paddy fields [17]. Several vector species multiply in a variety of biotopes and at different times of the year, adding another layer of complexity to the dynamics of entomological indices.

In order to avoid severe desiccation and heat stress during daytime, mosquitoes seek for resting habitats that provide a fresh and humid microclimate [18]. Daytime resting habitats have been identified both indoors (*e*.*g*. roof, wall, ceilings of houses and animal barns) and outdoors (*e*.*g*. tree holes, rodent holes, dense bushes, wells) [19]. We hypothesized that peridomestic dense bushes in and around the village are the main daytime resting habitat of *Anopheles* mosquitoes in Kayin state, and therefore proposed outdoor residual spraying (ORS) for malaria vector-control in this region. Several published studies have assessed the duration and magnitude of the residual insecticidal effects of insecticide mists applied to outdoor vegetation, and the subsequent impact on wild mosquito populations. Outcome measures included mosquito biting rate or abundance (assessed with human-landing catch and light traps respectively), oviposition [20, 21], recapture rate in mark-released experiments [22] and mortality of laboratory-adapted or wild female imagoes exposed to extemporaneously collected insecticide-treated plant material [21, 23, 24]. Both failure and success were reported with effects lasting from days to months. Most studies were conducted in North America and only three were for malaria vector-control in endemic areas [22, 25, 26]. After the studies on DDT in the 1940s [22, 25, 27], synthetic pyrethroids such as permethrin, deltamethrin, lambda-cyhalothrin and bifenthrin were preferred to other insecticide classes for their rapid knockdown effect and better toxicity profile [21, 23, 24, 28, 29]. ORS was carried out in a variety of settings ranging from urban backyards to hectares of jungle bush using backpack mist blowers, truck-mounted sprayers or aircrafts. Handling concentration and target dose of the active ingredient were usually that recommended on the product label. Gürtler *et al*. applied higher doses on peridomestic vegetation for the control of triatomine bugs that transmit Chagas disease in Argentina and did not report any adverse event to the spraying operators, by-standers or livestock [30, 31].

Recently, we determined the longevity of the insecticidal effect of pyrethroid mists applied to outdoor vegetation using a standard forced-contact assay and a pyrethroid-susceptible laboratory-adapted colony of *Anopheles dirus* [32]. Residual effects of a capsule suspension of lambda-cyhalothrin sprayed at a target concentration of 500 g a.i. /ha lasted for 42 and 98 days during the rainy and dry seasons respectively. The objective of the herein study was to assess ORS impact on the biting rate of malaria vectors in Kayin state, Myanmar.

## Methods

### Risk assessment

A detailed risk assessment was performed before the study in order to evaluate the risk of causing toxicity to human health, disrupting the ecosystem and selecting insecticide resistances in the mosquito vector populations, and to mitigate the negative effects of the spraying.

#### Use, toxicity and environmental fate of lambda-cyhalothrin

The insecticide chosen for the intervention was the 2.5% capsule suspension of lambda-cyhalothrin Karate Zeon1 2.5 CS (Syngenta, Basel, Switzerland). Lambda-cyhalothrin is a type II pyrethroid used for pest control in agriculture, forestry and public health. This insecticide was chosen because of the better toxicity profile of pyrethroids when compared to other insecticide classes, it is registered for outdoor use and it gives long-lasting insecticidal effects when applied to outdoor vegetation [32]. Outdoor application rate ranges between 7 and 540 g a.i. /ha and crop pre-harvest interval ranges between 1 and 90 days [33, 34]. Lambda-cyhalothrin is acutely toxic to mammals after ingestion, highly toxic after inhalation and it is of moderate toxicity upon contact with the skin. It is classified as unlikely to be genotoxic, carcinogenic or to cause reproductive or developmental effects. Lambda-cyhalothrin is highly toxic to aquatic organisms, terrestrial arthropods (including honeybees) but slightly to non-toxic to birds [33, 35]. Toxicity to reptiles and amphibians is likely high and overlooked [36, 37]. Lambda-cyhalothrin has a low vapour pressure, low solubility in water and high soil adsorption coefficient [35]. It is relatively photostable under natural irradiation. In the environment, lambda-cyhalothrin is degraded into inactive and non-toxic metabolites by photolysis, hydrolysis, microorganisms and plants [35]. Its fate in an ecosystem therefore depends on the nature of system components including soil, microbial community and plants, and of the climate. In surface water, the concentration of lambda-cyhalothrin decreases rapidly if suspended solids or aquatic organisms are present because lambda-cyhalothrin molecules are strongly adsorbed by particulates and plants. Some aquatic plants were also shown to rapidly assimilate and metabolise lambda-cyhalothrin dissolved in surface water [35]. Lambda-cyhalothrin is immobile in soils and the risk of leaching into streams and ground water is low [35]. A representative half-life in North American and European soils is 30 days with values ranging between 9 and 163 days [38, 39]. Temperature, humidity, aerobic microorganisms and plants are associated with shorter degradation time and probably contribute to the shorter half-lives reported in some tropical settings [35, 40, 41]. Lambda-cyhalothrin is not considered to be absorbed by the roots of terrestrial plants because it strongly binds to soil. When applied to vegetation, lambda-cyhalothrin can persist on foliage with half-life estimates ranging between 1.6 and 9.1 days [42], but values up to 40 days were reported [43]. The mechanisms of lambda-cyhalothrin clearance from plant surface are not known precisely. It certainly involves photolysis, hydrolysis and microorganisms on the plant surface, but uptake by leaves and plant metabolism may contribute.

#### Mitigation measures

In order to mitigate human health hazard and toxicity, the insecticide mist was not applied to human shelters and to plants used by local villagers for eating, drinking and preparing medicines or cosmetics. Moreover, the community engagement team of the Shoklo Malaria Research Unit did intensive preparatory work. The villagers and their headman were invited to participate in individual interviews, group discussion and workshops detailing no less than the exact nature of the study, and the implications, constraints, risks and benefits of the intervention. By-standers were asked to stay 100 meters or more from the operation and to wait 4 hours before entering into the treated area.

In order to mitigate toxicity to domestic animals and livestock, villagers shut it away under their house or outside of the village for 15 days after the intervention, and insecticide was not sprayed on plants used to feed livestock and on animal barns. In addition to domestic animals, we identified that aquatic organisms, wild mammals, amphibians, reptiles and terrestrial arthropods were the groups of non-target organisms most likely to be affected be the intervention because they are present in the sprayed area and lambda-cyhalothrin is toxic to those taxa. We were less concerned by the toxicity to birds because lambda-cyhalothrin is classified as slightly to non-toxic to birds, and most wild bird species nest in undisturbed habitats located at distance of the villages [44]. In order to mitigate toxicity to non-target organism, insecticide mist was not applied to flowering plants, beehives, and near streams. It is noteworthy that lambda-cyhalothrin is immobile in in soils and unlikely to leach into ground water. Given the timing of the intervention (beginning of the dry season) and the half-life of lambda-cyhalothrin in soils and foliage, most of the lambda-cyhalothrin was likely degraded before the next rains. Moreover, the insecticide mist was applied only once in two villages. Application of the insecticide mist was limited to some peridomestic vegetation in and around the village, it was not sprayed to large areas of wild nature. Hence, the spayed area was small in comparison to the size of the ecosystem.

Pyrethroid resistance was previously reported in malaria mosquito population collected in Kayin state [45]. In order to mitigate insecticide resistance in mosquitoes, insecticide mist was not applied around potential breeding sites thereby preventing insecticide leaching in water bodies where mosquito multiplies and subsequent exposure of mosquito larvae to sub-lethal concentration of lambda-cyhalothrin.

### Study sites

Four malaria-endemic villages in Kayin state were involved in the study based on their accessibility and willingness to participate in the study. The study villages, namely Klay Mo Khee (KM-500), Klay Poe Klo (KP-506), Mae Khel (MK-509) and Si Poe Khee (SP-513), were traditional Karen villages located on the foothills of the Dawna Range. The distance between the villages was 2.5 to 5 km. The land accessed is protected by the local Karen authorities. The villagers grow vegetable, beans, yam, fruit trees and sometime tobacco in the vicinity of their house whereas paddy fields cover large areas around the village. Villagers commonly own chickens, pigs and dogs that are kept below or near the house. Some families also own small goat flocks and rarely cows or buffaloes. The houses, built on silts, are made of wooden materials and roofed with leaves of antimony tree (*Dipterocarpus tuberculatus* Roxb), sometimes with sheet metal. The number of households was 62 in KM-500, 40 in KP-506, 27 in MK-509 and 38 in SP-613 (approximate population size between 100 and 300 persons /village).

### Intervention

ORS was carried out in two villages (MK-509 and SP-513) in December 2016 while the other two villages (KM-500 and KP-506) were used as a control. Outdoor residual spraying was carried out on 3^rd^ and 4^th^ December 2016 in MK-509, and on 5^th^ and 6^th^ December 2016 in SP-513. A capsule suspension of lambda-cyhalothrin (Karate zeon® 2.5% CS, Syngenta, Switzerland) was diluted at a concentration of 2 g a.i. /L with water, and sprayed on peridomestic dense bushes with a mist blower model PM7650H® (Makita, Anjo, Japan) at a target concentration of 500 g of a.i. /ha. This product was selected because it is widely used in agriculture and its formulation is optimized for lasting long on outdoor vegetation. The dose was set-up based on a literature review and risk assessment. The treated area covered a 20-meter wide band of dense bushes around the village and the peridomestic vegetation in between human shelters. No sensitive animals or plants were sampled.

### Entomological surveys

Malaria vectors were captured in all villages before and at one-month intervals for four months after the intervention (cool months of the dry season). Baseline surveys were conducted at the end of November (just before the intervention). Follow-up surveys started immediately after the intervention (beginning of December, month 0) and were carried out at one-month intervals until April (month 4). The survey schedule is presented in the S1 Appendix. All surveys were included in the analysis. Entomological surveys consisted of five consecutive nights of collection from 06:00 pm to 06:00 am in five houses per village and on one cow, as described previously [7]. In each village, five houses were randomly selected for mosquito sampling using the human-landing catch collection method. In each house, one mosquito collector sat indoors and one mosquito collector sat outdoors, yielding a total of 50 person-nights of collection per survey (25 person-nights indoors and 25 person-nights outdoors). Collectors were asked to catch every mosquito landing on their uncovered legs for 50 min per hour and allowed to rest for 10 min per hour. A cow-baited trap was also set-up yielding an additional five cow-nights of collection. One cow was isolated from the herd and a 1m-wide mosquito net was fenced around the animal, 30 cm above the ground level. One collector was asked to capture mosquitoes resting on the net for 50 min per hour and allowed to rest for 10 min. Mosquitoes were collected individually into 5 mL plastic tubes and shipped at the Shoklo Malaria Research Unit (Mae Sot, Thailand) at the end of each survey.

### Laboratory processing of the mosquito samples

Laboratory processing of the mosquito samples was performed as described previously [7]. Mosquitoes were identified by morphology at the genus level and *Anopheles* specimens were identified at the group level using the dichotomic key of Rattanarithikul *et al*. [46]. DNA was extracted from mosquito specimens using the cetyl-trimethylammonium bromide method [47]. Allele-specific polymerase chain reaction (AS-PCR) assays were used to discriminate *sensu stricto* species in the Funestus and Maculatus Groups [48, 49]. In case AS-PCR gave a negative result, and for the specimens in the Leucosphyrus Group, identification at the species level was performed by sequencing the internal transcribed spacer 2 (ITS2) DNA marker [50]. Insecticide resistances in malaria mosquitoes were assessed using wild-caught female *Anopheles* imagoes still alive at the end of the survey and a standard susceptibility assay [51].

#### DNA extraction

Whole mosquitoes were crushed individually in 200 μl of cetyl-trimethylammonium bromide solution 2% (TrisHCl pH = 8, 20mM; EDTA 10mM; NaCl, 1.4 mM; N-cetyl-N,N,N-trimethyl ammonium bromide 2%) with a TissueLyser II™ (Qiagen) set on 29 movements /second for 3 minutes. Samples were then warmed at 65°C for 5 minutes and 200 μl of chloroform were added. The aqueous phase was collected and DNA was precipitated with 200 μl of isopropanol. After centrifugation at 20,000 g for 15 minutes, the pellet was washed twice with 200 μl of 70% ethanol and suspended in 50 μl of PCR grade water.

#### Allele-specific polymerase chain reaction assays

The PCR mix was composed of 1X Goldstar™ DNA polymerase (Eurogentec, Seraing, Belgium) and 400 nM of each primer (Funestus assay: ITS2A 5’-TGT GAA CTG CAG GAC ACA T-3’, MIA 5’-CCC GTG CGA CTT GAC GA-3’, MIC 5’-GTT CAT TCA GCA ACA TCA GT-3’, ACO 5’-ACA GCG TGT ACG TCC AGT-3’, PAM 5’-TGT ACA TCG GCC GGG GTA-3’, VAR 5’-TTG ACC ACT TTC GAC GCA-3’; Maculatus assay: 5.8F 5’-TGT GAA CTG CAG GAC ACA T-3’, MAC 5’-CCC GTG CGA CTT GAC GA-3’, PSEU 5’-GTT CAT TCA GCA ACA TCA GT-3’, SAW 5’-ACA GCG TGT ACG TCC AGT-3’, K 5’-TGT ACA TCG GCC GGG GTA-3’, DRAV 5’-TTG ACC ACT TTC GAC GCA-3’). The PCR was conducted in a total reaction volume of 25 μl (1 μl of DNA template and 24 μl of PCR mix). The thermocycling protocol consisted in an initial activation step of 1 minute at 94°C, followed by 40 amplification cycles of 20 seconds at 94°C, 20 seconds at the appropriate annealing temperature (45°C and 55°C for the Funestus and Maculatus assays respectively), and 30 seconds at 72°C. The length of the PCR product was determined by gel electrophoresis in 2% agarose for 70 minutes at 120V.

#### Internal transcribed spacer 2 sequencing

Amplification of ITS2 was performed using the primer pair ITS2A (5'-TGT GAA CTG CAG GAC ACA T-3') and ITS2B (5'-ATG CTT AAA TTY AGG GGG T-3') described by Beebe and Saul [50]. The PCR mix was composed of 1X Goldstar™ DNA polymerase (Eurogentec, Seraing, Belgium) and 400 nM of each primer. The PCR was conducted in a total reaction volume of 25 μl (4 μl of DNA template and 21 μl of PCR mix). The thermocycling protocol consisted in an initial activation step of 1 min at 94°C, followed by 40 amplification cycles of 20 s at 94°C, 20 s at 51°C and 30 s at 72°C. The PCR product was sequenced by Macrogen™ (Seoul, South Korea) using the ITS2A primer. The sequence was analysed using the blastn algorithm of the online BLAST™ software in order to determine the corresponding species.

#### Insecticide susceptibility assay

Insecticide susceptibility tests were performed with alive mosquito specimens collected at the end of the follow-up (March and April surveys, *i*.*e*. three to four months after ORS intervention). Female imagoes still alive at the end of the surveys were identified at the group level by morphology. Specimens which belonged to the same taxa were introduced into standard plastic cylinders and exposed for 60 minutes to filter papers impregnated the 1X discriminating concentration of deltamethrin (0.05% or 18 mg of a.i. /m^2^), permethrin (0.75% or 275 mg of a.i. /m^2^) and lambda-cyhalothrin (0.05% or 18 mg of a.i. /m^2^) set by World Health Organization. Mosquitoes collected in different villages and/or during different surveys were tested independently. Mosquitoes collected in different sites (within the same village) and with different methods (human-landing catch or cow-baited trap) were pooled in the same test cylinders. The number of knocked down mosquitoes was recorded at the end of the exposure time. Mosquitoes were then transferred into standard holding tubes and provided with a 10% sugar solution. Mortality was recorded 24 hours after exposure to the insecticide. If sufficient number of collected specimens was available, mosquitoes exposed for 1 hour to a paper impregnated with the carrier (Dow 556 mixed with acetone) were used as a control. Tests were performed at 25 ± 2°C with a relative humidity of 70–80%. All insecticide testing materials used in this study were provided by the Vector Control Research Unit (VCRU), Universiti Sains Malaysia.

### Meteorological data

In this area, the rainy season usually starts in May and ends in November. Meteorological data were obtained from the Thai Meteorological Department, including daily records of rainfall and temperature in Mae Sot (Tak province) and Mae Sariang (Mae Hong Son province). The cumulative rainfall and mean temperature were calculated over a 15-day period before each collection date. Moon phase was taken into account in the analysis as a categorical variable with four levels (first quarter, full moon last quarter and new moon).

### Data analysis

Human-biting rate (HBR) and cow-biting rate (CBR) were defined as the number of collected mosquitoes divided by the number of person-nights or cow-nights respectively. The exophagy index (EI) was defined as the outdoor HBR divided by the sum of the indoor and outdoor HBRs. The cow-biting index (CBI) was defined as the CBR divided by the sum of the HBR and the CBR. Knockdown (KD) rate was defined as the number of knocked down mosquitoes divided by the number of exposed mosquitoes. Mortality rate was defined as the number of mosquitoes dead at the end of the 24-hour observation time divided by the number of exposed mosquitoes.

As expected, baseline characteristics of the two village pairs differed because of the small number of clusters (4 villages) and the non-randomized nature of the study (intervention was carried out in the villages with higher baseline HBR). In order to adjust for these differences, a propensity score for ORS intervention was generated with the survey (baseline and month 0–4), collection method (indoor or outdoor human-landing), mean temperature in the 15 days before the collection date, cumulative rainfall in the 15 days before the collection date and moon phase. ORS impact on the biting rate of malaria vectors was assessed using propensity scores adjusted generalized linear mixed effect regressions with a negative binomial distribution for the number of collected malaria vectors /collector /night (*i*.*e*. the unit of the model was one person-night of collection). Cow-baited trap collection method was not included in the analysis because the cow-biting rate did not follow the same distribution than human-biting rates and were not relevant to the understanding of ORS impact on human-vector contact. The variance was estimated with a robust method. We included two-level random effects (village and collection site) to account for nested dependency within the data. Parameter estimates may be non-robust to the failure of the assumed distribution of the random effects; therefore, diagnostic checking of the residuals was performed. As the data deviated from the assumption of equidispersion (average = variance) and from the dispersion parameter, it was evident that a negative binomial model was more appropriate. The Vuong test was performed to decide between zero inflated negative binomial and plain negative binomial model. The model was selected as best model with unique covariance structure for G-side random effects that produces the lowest Bayesian Information Criterion value. The covariance structures considered in the model were: AR(1) covariance structure, unstructured covariance structure, Toeplitz covariance structure and variance component structure.

### Ethics approval

The protocol for mosquito collection and analysis has been approved by the Oxford Tropical Research Ethics Committee (1015–13, dated 29 Apr 2013), by the Ethics Review Committee for Research Involving Human Research Subjects, Health Science Group, Chulalongkorn University (COA 154/2014), by the Karen Department of Health and Welfare, Karen National Union and by the Tak Province Border Community Ethics Advisory Board [52]. All participants provided their written consent to participate in this study. This consent procedure was approved by the ethics committees.

## Results

### Baseline assessment of *Anopheles* diversity, blood feeding behavior and biting rates

Two thousand one hundred forty-seven *Anopheles* specimens were collected during baseline surveys (200 person-nights and 20 cow-nights of collection) and identified by morphology. Moreover, 97% (616/636), 98% (1350/1375) and 93% (13/14) of the specimens in the Funestus, Maculatus and Leucosphyrus Groups respectively were identified with molecular assays. We report the occurrence of five *Anopheles* species *sensu lato* and ten *Anopheles* species *sensu stricto* including: *An*. *annularis* (*s*.*l*.) (Annularis Group), *An*. *barbirostris* (*s*.*l*.) (Barbirostris Group), *An*. *baimaii* (*s*.*s*.) (Leucosphyrus Group, Dirus Complex), *An*. *hyrcanus* (*s*.*l*.) (Hyrcanus Group), *An*. *jamesii* (*s*.*l*.) (Jamesii Group), *An*. *kochi* (Kochi Group), *An*. *maculatus* (*s*.*s*.), *An*. *sawadwongporni*, *An*. *pseudowillmori* (Maculatus Group), *An*. *minimus* (*s*.*s*.), *An*. *harrisoni* (Funestus Group, Minimus Complex), *An*. *jeyporiensis* (Funestus Group), *An*. *subpictus* (*s*.*l*.) (Subpictus Group), *An*. *tessellatus* (Tessellatus Group) and *An*. *karwari* (undefined Group). The most abundant human-biting species were *An*. *minimus* (*s*.*s*.), *An*. *maculatus* (*s*.*s*.), *An*. *pseudowillmori* and *An*. *sawadwongporni* (Table 1).

**Table 1:** Village-collated mean estimates of mosquito human-biting rates determined during baseline surveys.

Genus	Group	Species	Mean human-biting rate estimate of the taxon in the indicated village expressed in bites /person /night [range]			
			KM-500 (Nov 30^th^ to Dec 4^th^) ^a^	KP-506 (Nov 29^th^ to Dec 3^rd^) ^a^	MK-509 (Dec 1^st^ to Dec 5^th^) ^a^	SP-513 (Nov 29^th^ to Dec 3^rd^) ^a^
*Aedes*	**-**	spp.	5.98 [0–37]	0.32 [0–3]	5.74 [0–36]	0.86 [0–8]
*Culex*	**-**	spp.	0.28 [0–2]	0.12 [0–2]	1.94 [0–12]	3.24 [0–24]
*Anopheles*	**-**	spp.	1.26 [0–12]	0.98 [0–7]	6.68 [0–44]	20.1 [0–128]
	Annularis	spp. ^b^	0 [0–0]	0 [0–0]	0.04 [0–1]	0.18 [0–3]
	Barbirostris	spp. ^b^	0 [0–0]	0.02 [0–1]	0 [0–0]	0.1 [0–2]
	Funestus	spp.	0.64 [0–8]	0.14 [0–3]	2.6 [0–17]	5.16 [0–35]
		*An*. *minimus* (*s*.*s*.) ^c^	0.62 [0–7]	0.14 [0–3]	2.54 [0–17]	4.86 [0–34]
		*An*. *harrisoni*	0 [0–0]	0 [0–0]	0 [0–0]	0.02 [0–1]
		*An*. *jeyporiensis*	0 [0–0]	0 [0–0]	0.02 [0–1]	0 [0–0]
	Hyrcanus	spp. ^d^	0 [0–0]	0 [0–0]	0.08 [0–1]	0.1 [0–1]
	Jamesii	spp. ^d^	0 [0–0]	0 [0–0]	0.02 [0–1]	0.08 [0–2]
	Kochi	spp.	0 [0–0]	0 [0–0]	0.12 [0–3]	0.02 [0–1]
	Leucosphyrus	spp.	0.04 [0–1]	0 [0–0]	0.12 [0–1]	0.06 [0–1]
		*An*. *baimaii* ^c^	0.04 [0–1]	0 [0–0]	0.12 [0–1]	0.04 [0–1]
	Maculatus	spp.	0.58 [0–4]	0.82 [0–7]	3.68 [0–28]	14.34 [0–90]
		*An*. *maculatus* (*s*.*s*.) ^c^	0.22 [0–2]	0.02 [0–1]	0.9 [0–14]	3.14 [0–21]
		*An*. *pseudowillmori* ^b^	0.26 [0–4]	0.76 [0–6]	2.56 [0–16]	10.42 [0–69]
		*An*. *sawadwongporni* ^c^	0.1 [0–2]	0 [0–0]	0.18 [0–6]	0.6 [0–3]
	Subpictus	spp. ^d^	0 [0–0]	0 [0–0]	0 [0–0]	0 [0–0]
	Tessellatus	spp. ^d^	0 [0–0]	0 [0–0]	0 [0–0]	0 [0–0]
	Unclassified	*An*. *karwari* ^d^	0 [0–0]	0 [0–0]	0.02 [0–1]	0 [0–0]

^a^ each survey consisted of 25 person-nights of collection indoors and 25 person-nights of collection outdoors (total of 50 person-nights /village), the numbers of mosquitoes collected indoors and outdoors were pooled to calculate the mean human-biting rate.

^b^ secondary malaria vectors.

^c^ primary malaria vectors.

^d^ some species in these Groups are efficient malaria vectors elsewhere, but were never found infected with human malaria parasites on the Thailand-Myanmar border (*e*.*g*. *An*. *karwari*, *An*. *sinensis*, *An*. *subpictus* (*s*.*s*.), *An*. *splendidus* and *An*. *tessellatus*) [7].

**Fig 1:**
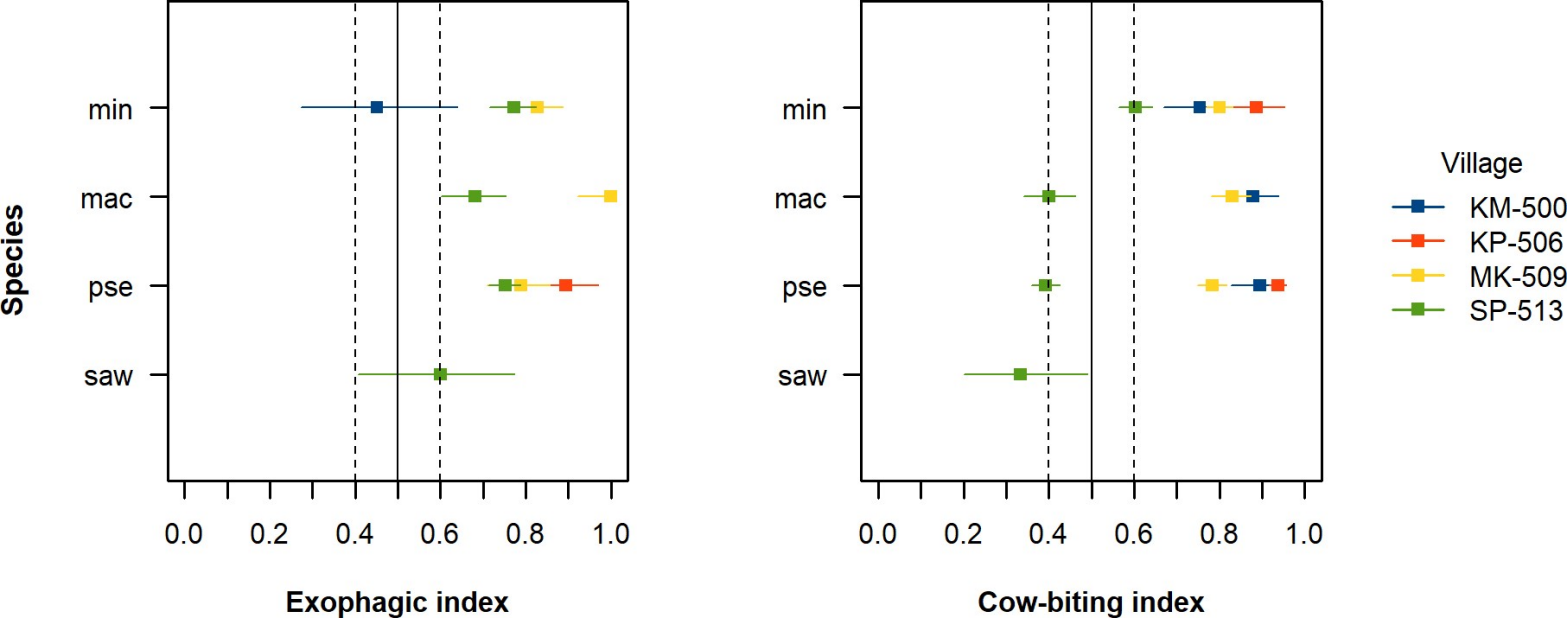
Exophagic and cow-biting index estimates of malaria mosquitoes at baseline. EI and CBI were not estimated if the total number of collected mosquitoes was less than 15 for the corresponding index. Error bars indicate exact binomial 95% CIs. *Abbreviations*: CBI, cow-biting index; CI, confidence interval; EI, exophagic index, pse: *An*. *pseudowillmori*, mac, *An*. *maculatus* (*s*.*s*.) min, *An*. *minimus* (*s*.*s*.), saw: *An*. *sawadwongporni*.

**Fig 2:**
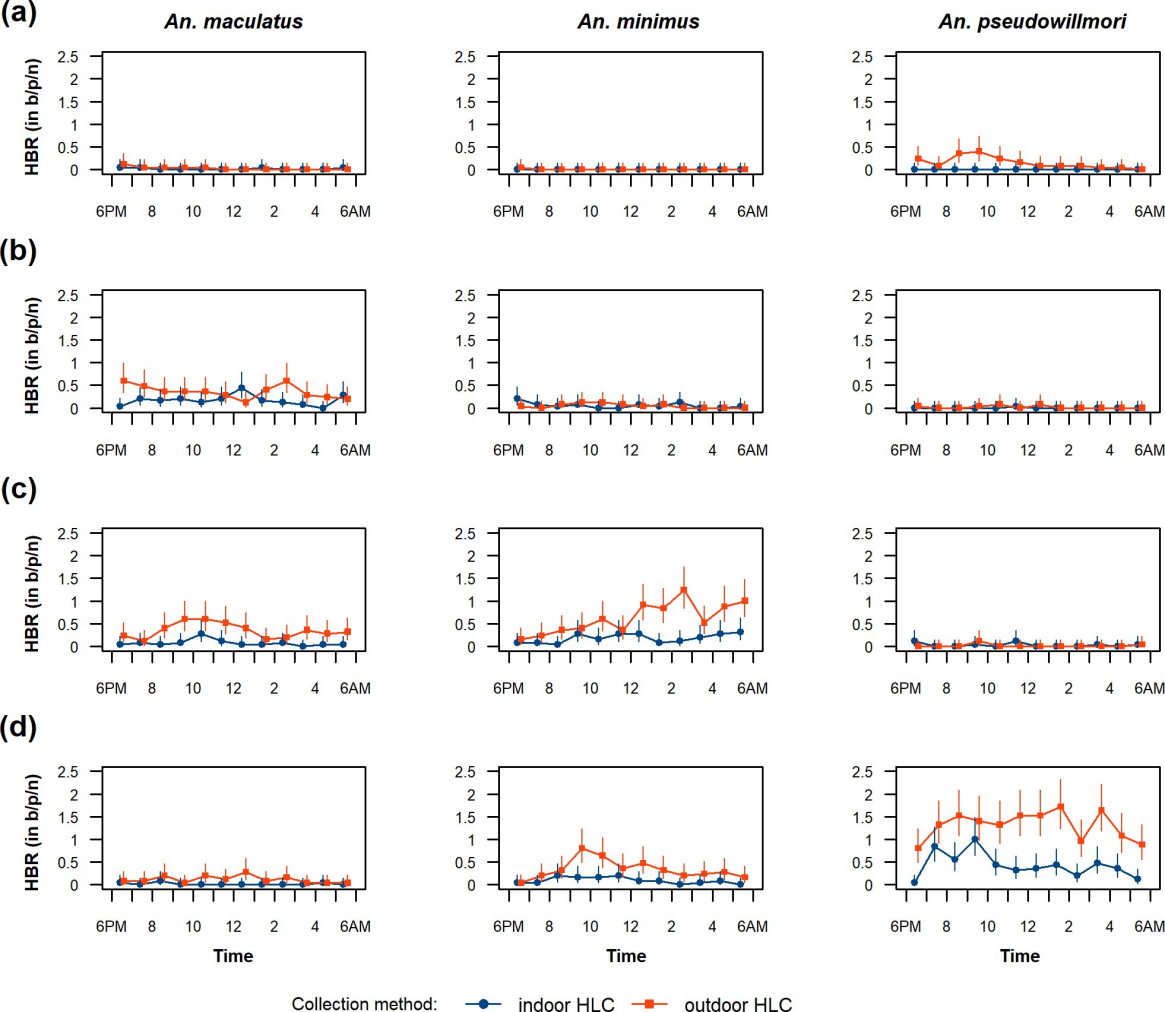
Hourly biting pattern of malaria mosquitoes at baseline. (a) KM-500. (b) KP-506. (c) MK-509. (d) SP-513. Error bars indicate exact Poisson 95% CIs. *Abbreviations*: b/p/n: bites /person /night; CI, confidence interval; HLC, human-landing catch.

Malaria vectors were predominantly exophagic and zoophagic with village-collated EI and CBI estimates ranging from 0.45 to 1.00 and from 0.33 to 0.95 respectively (Fig 1). The analysis of hourly biting pattern showed activity peaks during the early evening and early morning (Fig 2). Only 24% (339/1409) of the specimens in the Funestus, Maculatus and Leucosphyrus Groups were collected indoors between 9 PM and 5 AM. EI, CBI and hourly biting pattern of a given species were significantly different from one village to another, hence suggesting the importance of local ecological factors in shaping the blood seeking behaviour of malaria mosquitoes.

When taking into account only the specimens in the Funestus, Maculatus and Leucopshyrus Groups, the proportion of human-landing catches with a positive count of mosquito vectors varied between 16% and 96% according to the village. Village-collated mean HBR estimates were consistently higher than the corresponding median and unaggregated HBR measurements ranged between 0 and 125 bites /person /night. Village-collated mean CBR estimates varied between 12.2 and 50.4 bites /cow /night and unaggregated measurements ranged between 3 and 81 bites /cow /night (Table 2).

**Table 2:** Descriptive statistics of malaria vector biting rates at baseline.

**Biting rate**	**Parameter**	**Parameter estimate in the indicated village**			
		**KM-500**	**KP-506**	**MK-509**	**SP-513**
indoor HBR (in bites/person/night)	p (in %) ^a^	52	16	68	80
	minimum	0	0	0	0
	10^th^ percentile	0	0	0	0
	median	1	0	2	3
	mean	1.28	0.2	2.12	9.96
	90^th^ percentile	2.6	1	4.6	30.8
	maximum	12	2	10	38
outdoor HBR in bites /person/night)	p (in %)	52	64	92	96
	minimum	0	0	0	0
	10^th^ percentile	0	0	1	1.4
	median	1	1	5	17
	mean	1.24	1.72	10.68	29.16
	90^th^ percentile	2	4	25.8	83
	maximum	9	7	42	125
CBR (in bites/cow/night)	p (in %)	100	100	100	100
	minimum	3	14	26	13
	10^th^ percentile	5	16.4	26.4	20.2
	median	13	28	38	40
	mean	12.2	26	50.4	34.6
	90^th^ percentile	19.4	34.8	80.6	45
	maximum	23	38	81	47

*Abbreviations*: CBR, cow-biting rate; HBR, human-biting rate.

^a^ p, proportion of catches with positive counts of malaria vectors expressed as a percentage. Only the specimens in the Funestus, Maculatus and Leucopshyrus Groups were taken into account for parameter estimations.

### Impact of outdoor residual spraying on malaria vector human-biting rates

**Fig 3:**
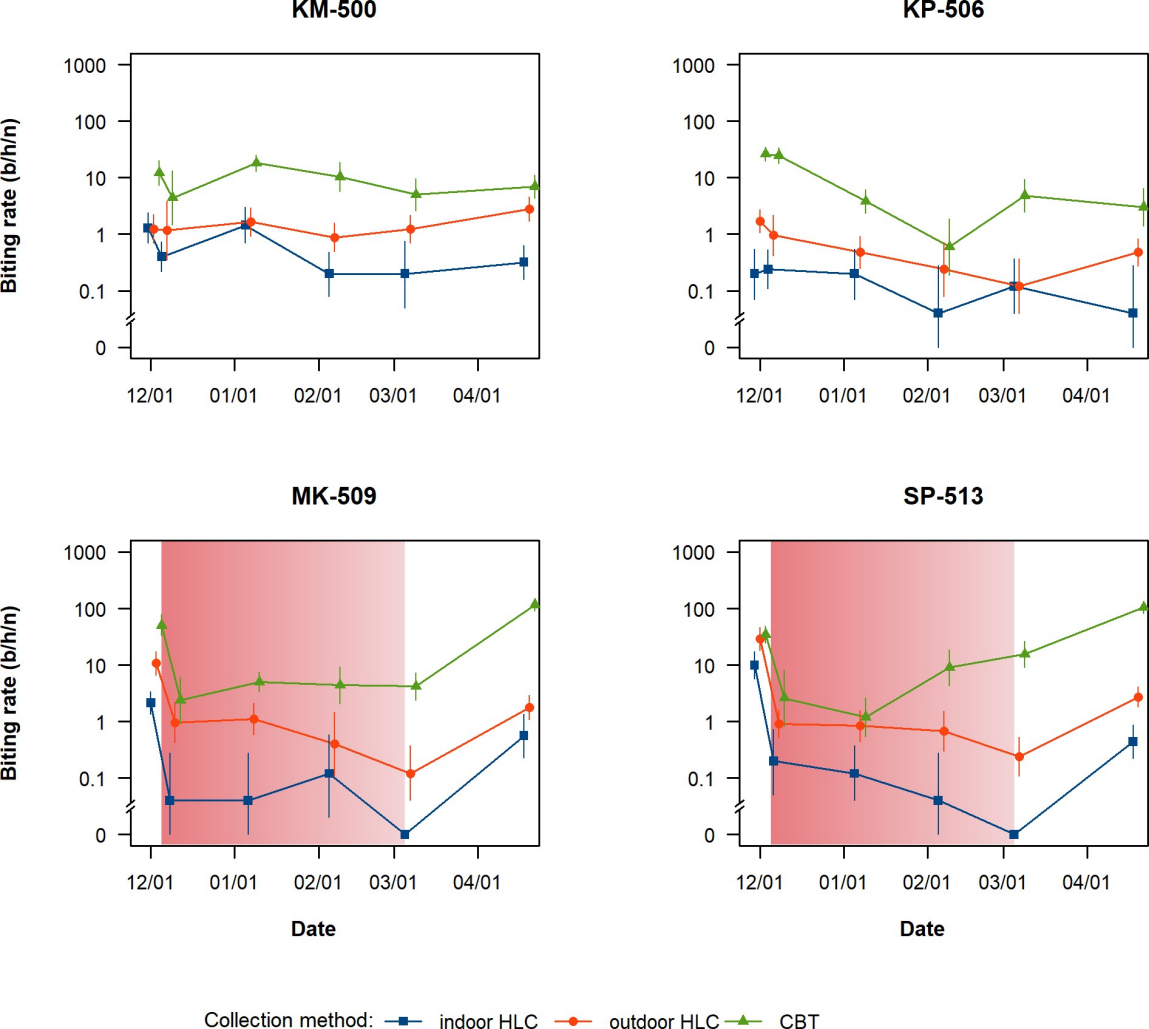
Impact of ORS on malaria vector biting rates. ORS intervention was carried out on 3^rd^ and 4^th^ December 2016 in MK-509 and on 5^th^ and 6^th^ December 2016 in SP-513. Gradient-filled panels show previous estimate of the longevity of the residual insecticidal effects of lambda-cyhalothrin mist applied to outdoor vegetation (98 days) [32]. Error bars indicate the negative binomial regression 95% confidence intervals. *Abbreviations*: b/h/n: bites /host /night; CI, confidence interval; HBR, human-biting rate; ORS, outdoor residual spraying.

Only the specimens in the Funestus, Maculatus and Leucopshyrus Groups were taken into account for assessing the impact of ORS on the biting rates of malaria mosquitoes. Including other *Anopheles* species in the analysis did not change the results. The observed biting rates of malaria mosquitoes are presented in the Fig 3 and S1 Appendix. Village-collated mean biting rate estimates at baseline were higher in the intervention villages than in the controls and drastically decreased in the intervention villages immediately after ORS. The effect was observed indoors, outdoors and in the cow-baited trap. The HBR remained low during 3 months after ORS in all villages and rose again at month 4 in the intervention villages but not in the controls. The same trend was observed in the cow-baited traps except in SP-513 (intervention village) where the CBR increased gradually after 2 months.

Using model outputs, we estimated that HBR in the sprayed villages was 9.6 times higher than that in the controls (95%CI = 5.7–16.5, P <0.0001), and that outdoor HBR was 4.1 times higher than indoor HBR (95%CI = 3.35–5.10, P = <0.0001). HBR naturally declined after baseline survey with incidence rate ratio (IRR) estimates varying between 0.26 (95%CI = 0.14–0.48, P<0.0001) and 0.67 (95%CI = 0.34–1.30, P = 0.2364) during the follow-up. HBR was reduced by 91% (95%CI = 88–96, P <0.0001) immediately after ORS. In the sprayed villages, the HBR remained lower during the entire follow-up when compared to baseline with IRR ranging between 0.02 (95%CI = 0.01–0.06, P <0.0001) and 0.19 (95%CI = 0.09–0.40, P <0.0001) at month 3 and month 4 respectively (Table 3).

**Table 3:** Generalized linear mixed-effect model output for the multivariable analysis of the number of collected malaria vectors /person /night including location, visits and outdoor residual spraying as predictors.

Variable	Category	Estimate (SE)	IRR (95%CI)	p-value ^a^
Study arm	Control (reference)	0	1	-
	ORS	2.3 (0.27)	9.66 (5.66–16.50)	<0.0001
Collection method	indoor HLC (reference)	0	1	-
	outdoor HLC	1.4 (0.11)	4.13 (3.35–5.10)	<0.0001
Survey	baseline (reference)	0	1	
	month 0	-0.8 (0.32)	0.45 (0.24–0.84)	0.0124
	month 1	-0.4 (0.34)	0.67 (0.34–1.30)	0.2364
	month 2	-1.4 (0.32)	0.26 (0.14–0.48)	<0.0001
	month 3	-1.2 (0.32)	0.31 (0.17–0.58)	0.0003
	month 4	-0.4 (0.29)	0.64 (0.36–1.14)	0.1269
Survey*Arm	baseline:ORS	0	1	-
	month 0:ORS	-2.4 (0.46)	0.09 (0.04–0.22)	<0.0001
	month 1:ORS	-2.7 (0.44)	0.06 (0.03–0.15)	<0.0001
	month 2:ORS	-2.5 (0.49)	0.08 (0.03–0.21)	<0.0001
	month 3:ORS	-3.8 (0.48)	0.02 (0.01–0.06)	<0.0001
	month 4:ORS	-1.7 (0.39)	0.19 (0.09–0.40)	<0.0001

*Abbreviations*: CI, confidence interval; HLC, human-landing catch; IRR, incidence rate ratio; ORS, outdoor residual spraying.

^a^ The P-value was calculated from mixed effect negative binomial regression model after adjusting for propensity scores.

### Insecticide resistance

A subsample of 552 specimens collected during March and April surveys were used to perform insecticide susceptibility tests, of which 434 were exposed to insecticides and 118 were used for controls (Table 4 and S2 Appendix). Given the diversity of *Anopheles* mosquito species and the relatively low biting rates, most of the tests were performed with small number of mosquitoes and without control. The number of exposed *An*. *maculatus* (*s*.*l*.) and *An*. *minimus* (*s*.*l*.) was large enough to estimate mortality with reasonable certainty. Mortality rate of permethrin, deltamethrin and lambda-cyhalothrin against *An*. *maculatus* (*s*.*l*.) was 97, 95 and 87% respectively. Mortality rate of lambda-cyhalothrin against *An*. *minimus* (*s*.*l*.) was 91%. The KD rate at the end of the exposure time ranged between 85 and 91%. Only one out of eight *An*. *barbirostris* (*s*.*l*.) was dead at the end of the tests. Insecticide resistant mosquitoes were detected in all villages.

**Table 4:** Results of the standard suceptibility tests performed with wild-caught female imagoes using deltamethrin, lambda-cyhalothrin (“0.05%” or 18 mg of a.i. /m^2^) or permethrin (“0.75%” or 275 mg of a.i. /m^2^).

Species	Insecticide	No. exposed mosquitoes	No. knocked-down mosquitoes after 60 min	No. dead mosquitoes after 24 hours	KD60 in % (95%CI) ^a^	Mortality in % (95%CI) ^a^
*An*. *barbirostris* (*s*.*l*.)	lambda-cyhalothrin	8	0	1	0 (0–37)	12 (0–53)
*An*. *dirus* (*s*.*l*.)	lambda-cyhalothrin	1	0	1	0 (0–98)	100 (3–100)
*An*. *jamesii* (*s*.*l*.)	lambda-cyhalothrin	20	19	18	95 (75–100)	90 (68–99)
*An*. *kochi* (*s*.*s*.)	lambda-cyhalothrin	1	1	1	100 (3–100)	100 (3–100)
*An*. *maculatus* (*s*.*l*.)	deltamethrin	102	89	97	87 (79–93)	95 (89–98)
	lambda-cyhalothrin	163	144	142	88 (82–93)	87 (81–92)
	permethrin	58	53	56	91 (81–97)	97 (88–100)
*An*. *minimus* (*s*.*l*.)	lambda-cyhalothrin	88	75	80	85 (76–92)	91 (83–96)

*Abbreviations*: CI, confidence interval; KD60, knockdown rate at the end of the 60 min period of exposure to insecticide.

^a^ 95% binomial confidence intervals were calculated for KD60 and mortality rates.

## Discussion

To our knowledge, this is the first assessment of ORS for malaria vector-control in a Southeast Asian transmission setting since Nair studies on DDT in the 1940s [25]. We estimated that the HBR of primary malaria vector species was divided by 91% (95%CI = 88–96, P <0.0001) immediately after applying a lambda-cyhalothrin mist to outdoor vegetation, thereby strongly suggesting that peridomestic dense bushes in and around the village are an important resting site of malaria mosquitoes in this area. Interestingly, the HBR decreased both indoors and outdoors. This result confirms that mosquito vectors seeking a blood meal indoors spend most of their life cycle outside. As expected, there was a rich diversity of malaria mosquito species in the area of the study [7, 53]. This diversity translates into complex transmission dynamics and challenges the efficacy malaria vector-control with mosquito bed nets, indoor residual spraying or larval source management [8, 16, 54, 55]. Therefore, the impact of ORS on multiple malaria mosquito vector species is an important feature of this intervention.

The impact of ORS on mosquito biting rate may involve several mechanisms. The sharp decrease in biting rates immediately after the intervention implies that an important proportion of the adult population was affected by the intervention. At the concentration used in this study, lethal effect is to be expected if insecticide mist reaches resting mosquitoes, thus ORS may result in mass killing of the vector population. In addition, pyrethroids have irritant and excito-repellent properties that deter mosquitoes and compete with the lethal effect during operational deployment of vector-control interventions [56]. This mechanism may also lower mosquito density inside the village after the spraying, especially when using lambda-cyhalothrin which is irritant to susceptible and resistant mosquitoes [57]. The rise in malaria vector abundance observed 4 months after ORS in the intervention villages but not in the controls coincided with the expected end of the residual effects of this insecticide formulation applied to outdoor vegetation [32]. This result suggests the importance of the residual effects in determining intervention efficacy. Noteworthy, both male and female *Anopheles* mosquitoes rest on outdoor vegetation [18] and intervention impact on male mosquitoes is likely to be an important factor.

There were several limitations to this study. We did not collect data on the resting sites, residual effect and biting rate outside of the intervention area because of the logistic constrains it would have implied and the pilot nature of the study, therefore it is not possible to draw conclusion on the mechanism of action of ORS. Moreover, the intervention was not randomized (villages with higher HBR estimates at baseline were selected for ORS) and only two pairs of villages were included in this pilot study. Given the small number of clusters and the timing of ORS intervention (transition period during the rainy and the dry seasons, when vector populations naturally declines), it was not possible to determine precisely the duration and magnitude of ORS impact on malaria vector biting rate. We did not assess intervention effects on transmission intensity and malaria incidence. Falciparum malaria has been eliminated from these villages before the study [2] and the incidence of symptomatic vivax malaria is mostly driven by relapses [58], thereby confounding the relationship between malaria incidence and the *P*. *vivax* entomological inoculation rate. Importantly, vector longevity is a key parameter in the equation of vectorial capacity [59, 60] and should also be used as an outcome measure in future studies. The toxicity of the insecticide mist to non-target organisms is likely to be high given the nature of sprayed sites. Although we did not collect data, the villages were closely monitored during and after the intervention by the study team and villagers. We did not observe any dramatic impact of the intervention on the ecosystem (especially on the taxa at high risk including aquatic organisms, wild mammals, amphibians, reptiles and terrestrial arthropods). Noteworthy, the risk that insecticide residues were washed into streams when the rains came was deemed very low given the high soil adsorption coefficient of lambda-cyhalothrin, its half-life in soils, the timing of the intervention (beginning of the dry season) and that insecticide mist was not applied near streams.

Deltamethrin and permethrin resistances were reported in Kayin state and may pose an additional challenge to effective vector-control intervention in this region [45]. We did not perform insecticide susceptibility tests during baseline surveys precluding the assessment of ORS effect on resistance dynamics. Mortality rates were lower with lambda-cyhalothrin (type II pyrethroid) than with deltamethrin and permethrin (type I pyrethroids). Type II pyrethroids are intensively used for agriculture in this region [61]. This observation suggests that agriculture may play an important role in the emergence and/or selection of resistance to insecticides used in public health. The KD rate after 60 min of exposure to insecticide was high in *An*. *minimus* (*s*.*l*.) and *An*. *maculatus* (*s*.*l*.) suggesting the involvement of metabolic pathways rather than *kdr* mutations in these species.

## Conclusions

Outdoor residual spraying with a capsule suspension of lambda-cyhalothrin rapidly decreases exophilic malaria vector biting rates in this area where pyrethroid resistance has been documented.

## Supporting information

S1 Appendix Raw data of mosquito catches. (XLSX)

S2 Appendix Raw data of the insecticide susceptibility tests. (XLSX)
